# Supplementary figures and images for: A variational autoencoder trained with priors from canonical pathways increases the interpretability of transcriptome data
Source: PLoS Comput Biol. 2024 Jul 3;20(7):e1011198. doi: 10.1371/journal.pcbi.1011198 (PMC11251626; doi:10.1371/journal.pcbi.1011198)

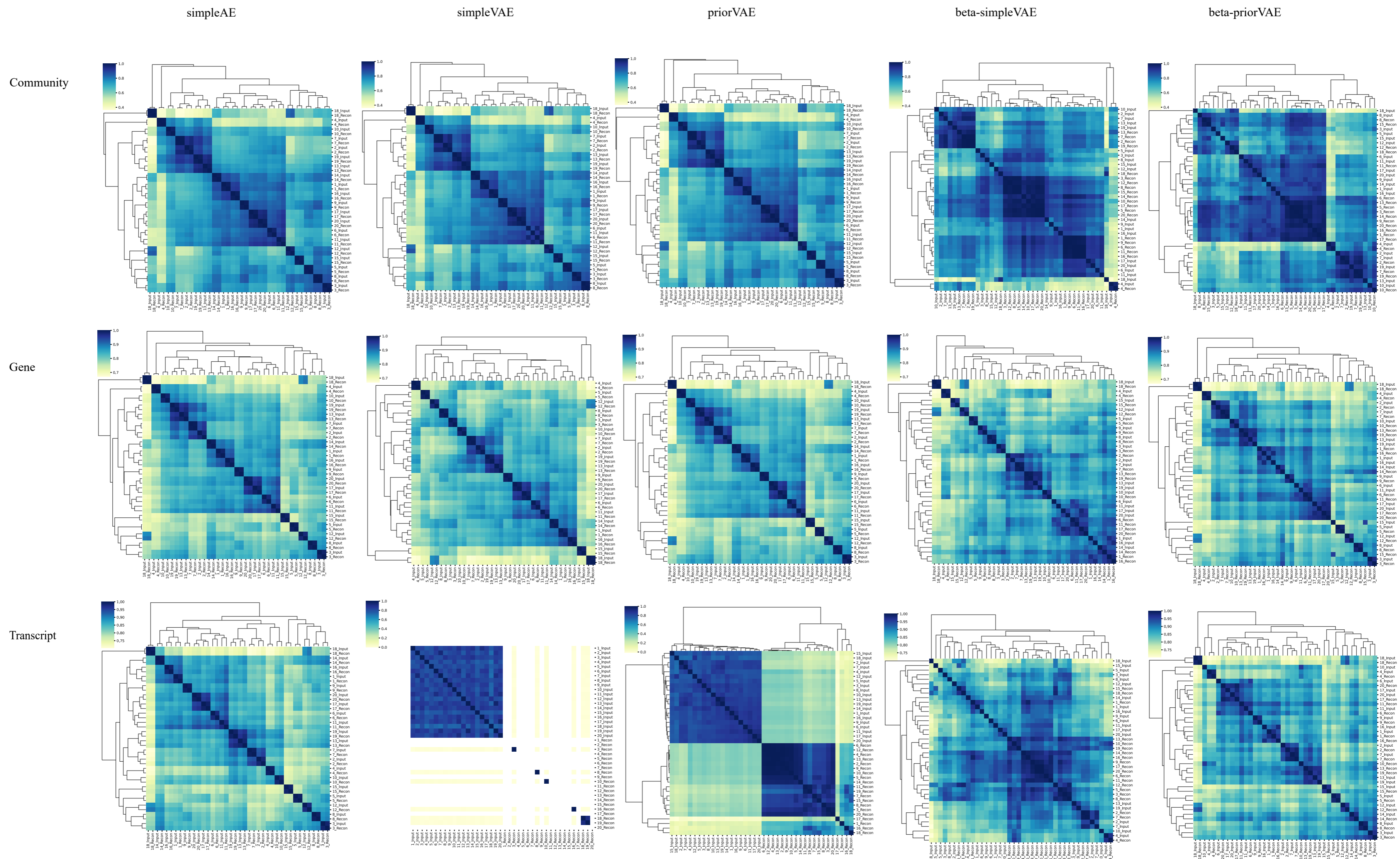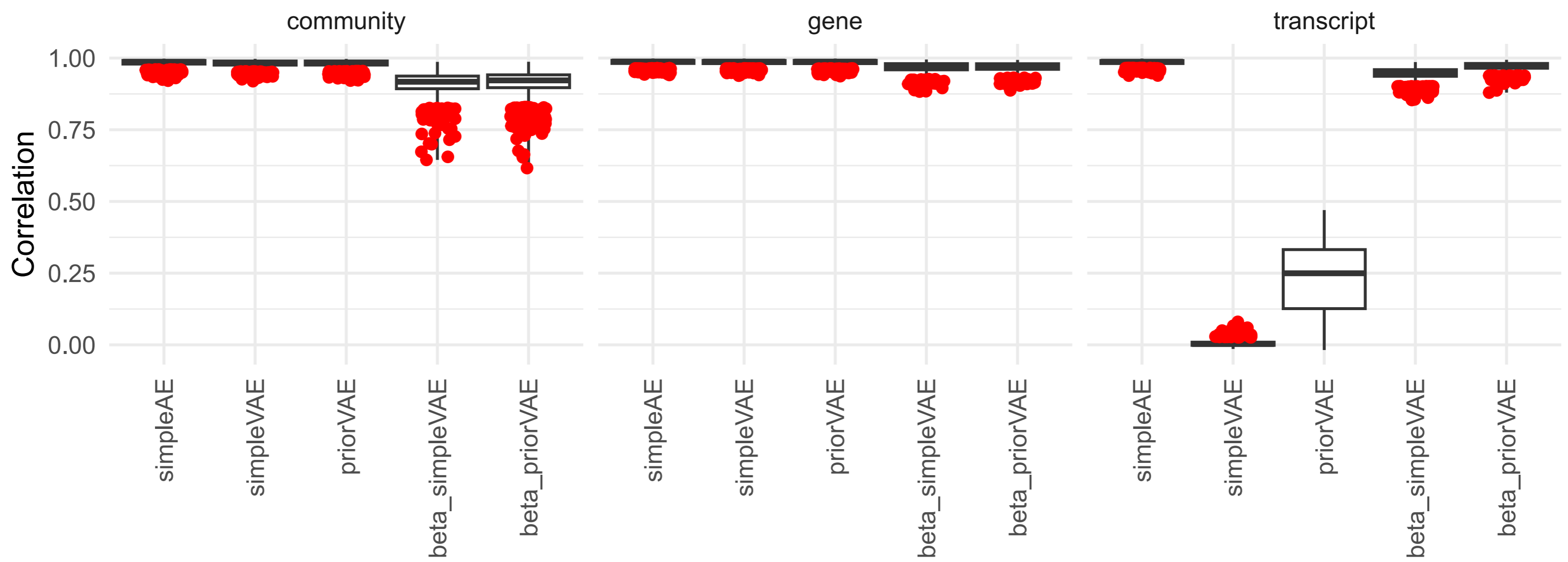

Supplement: S1 Fig — The pairwise correlation plots include both input and output transcriptomes. The Boxplots depict the correlations between every input and their respective output. (PDF) [file pcbi.1011198.s001.pdf]

A

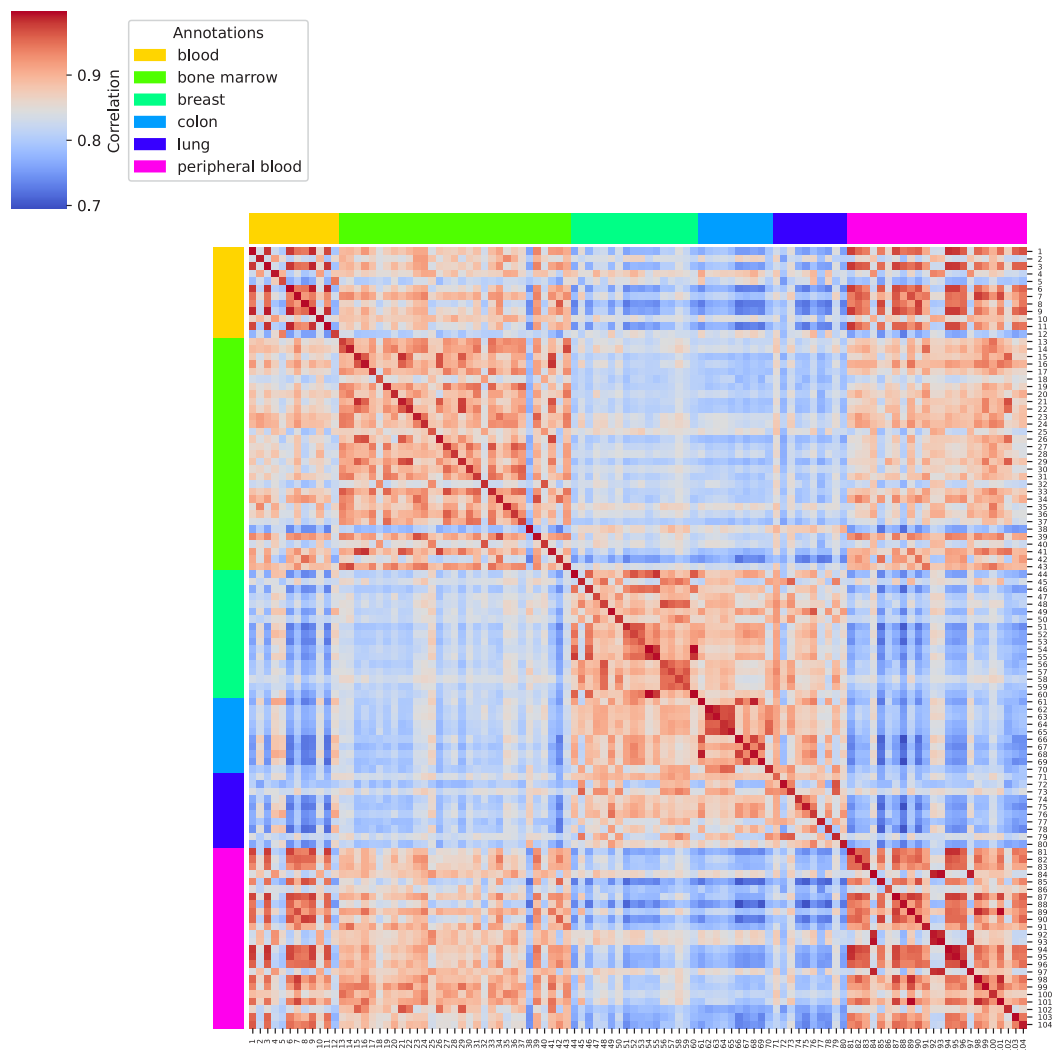

B

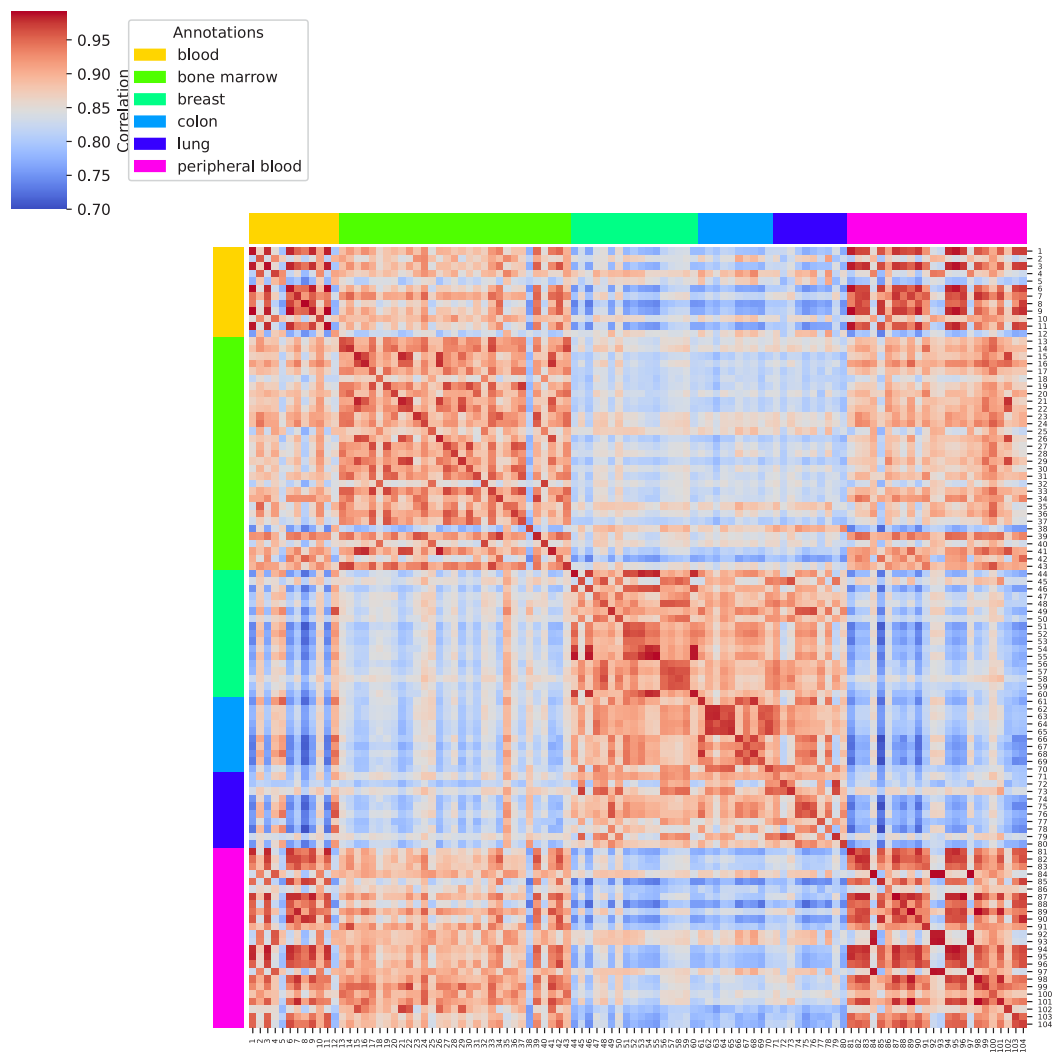

Supplement: S2 Fig — The rows and columns are ordered by sample, so the diagonal reports the correlation for the input-output pair for one sample. (PDF) [file pcbi.1011198.s002.pdf]

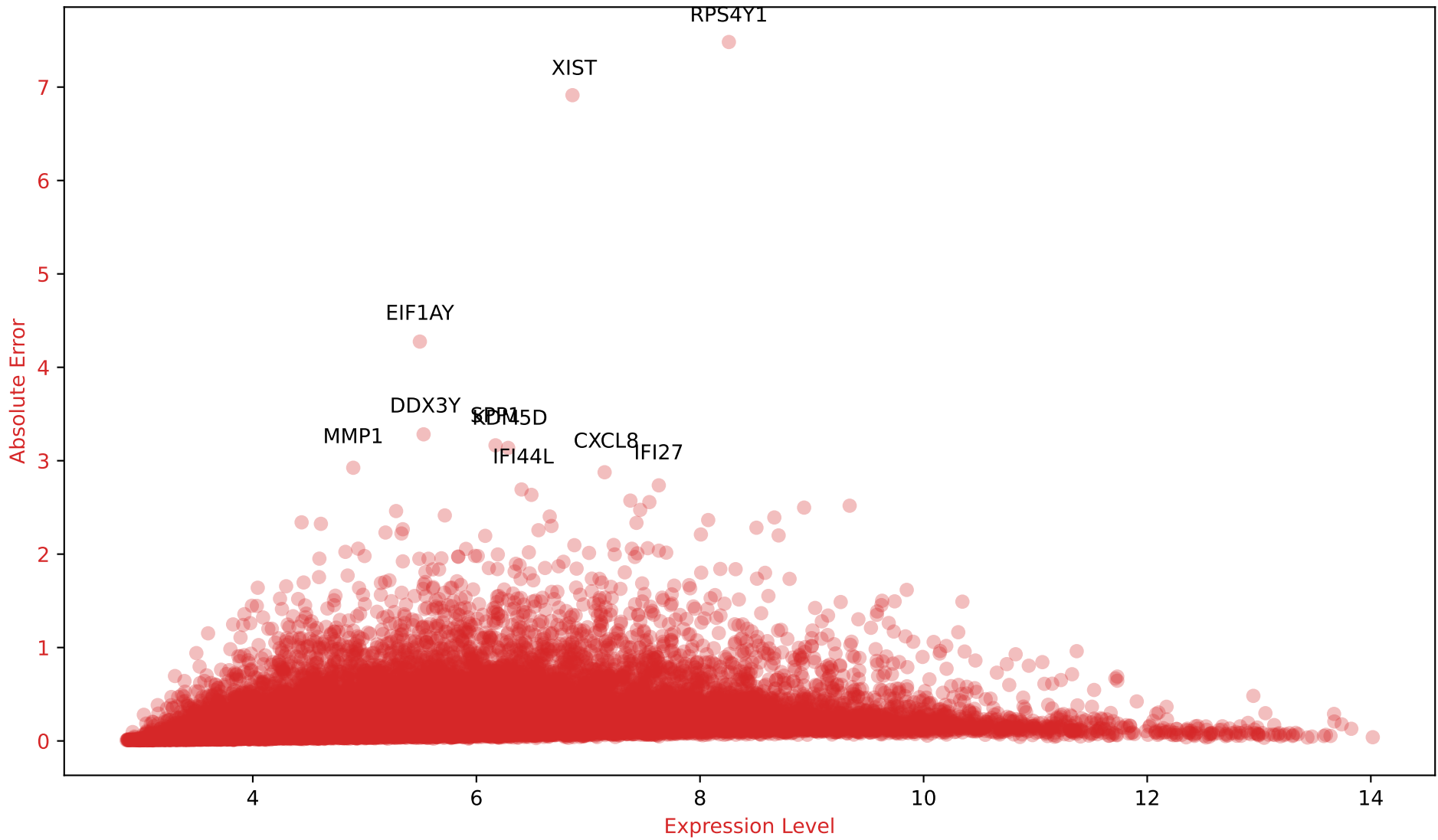

Supplement: S3 Fig — The absolute error between input and output gene levels was calculated for each gene and plotted as a function of the average expression level. (PDF) [file pcbi.1011198.s003.pdf]

A

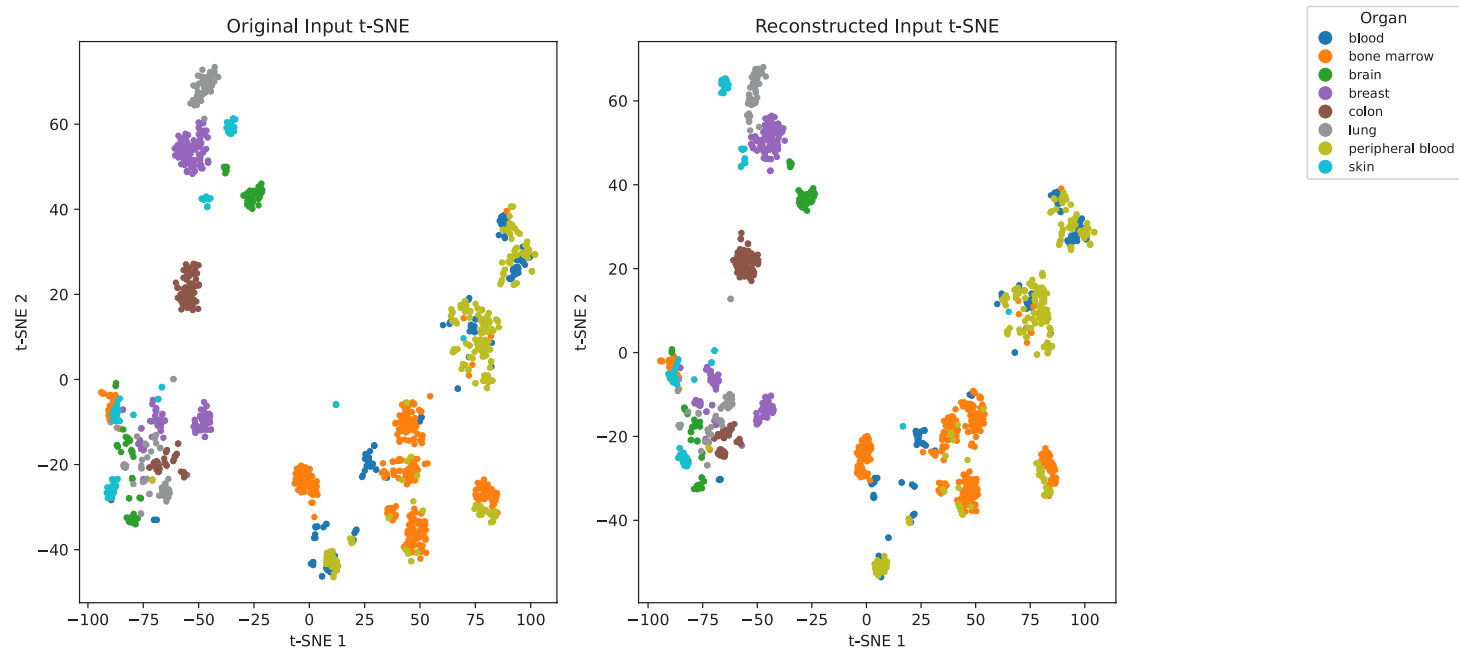

B

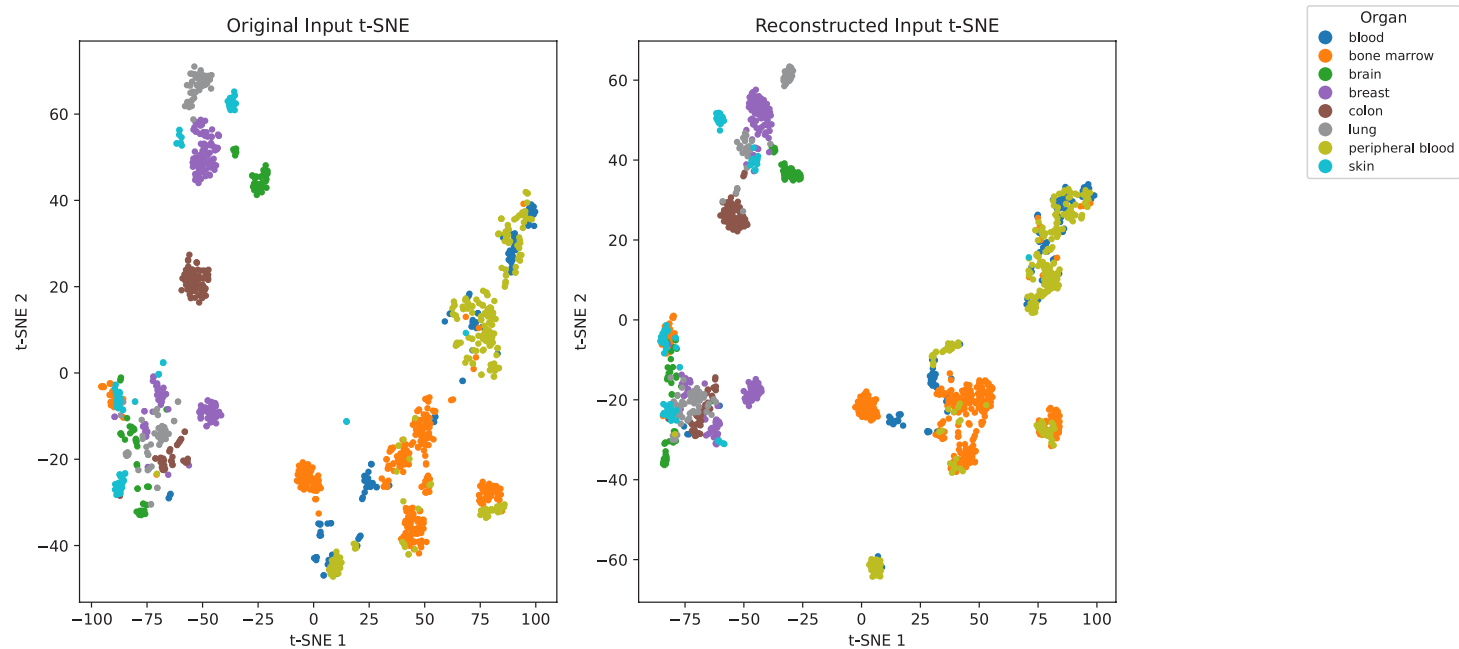

Supplement: S4 Fig — A Shows the performance for the prior VAE and B for beta-prior VAE. (PDF) [file pcbi.1011198.s004.pdf]

**A**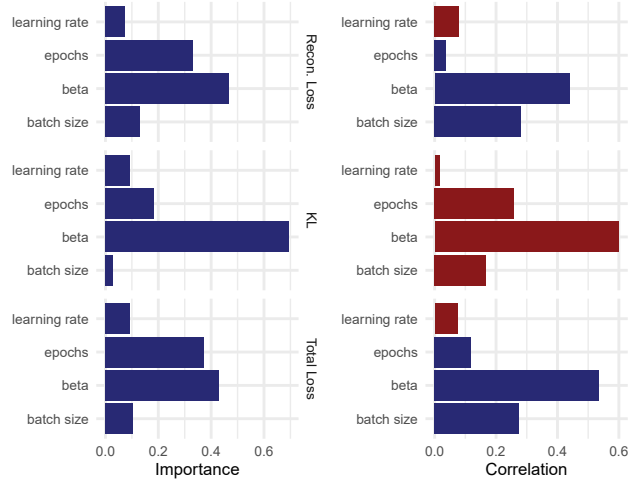**B**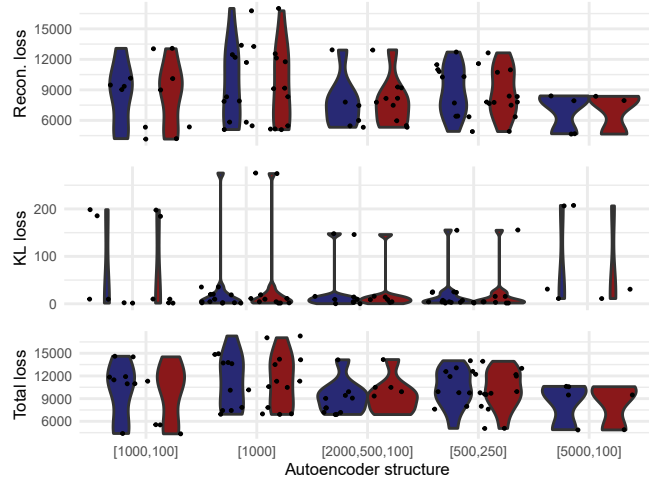**C**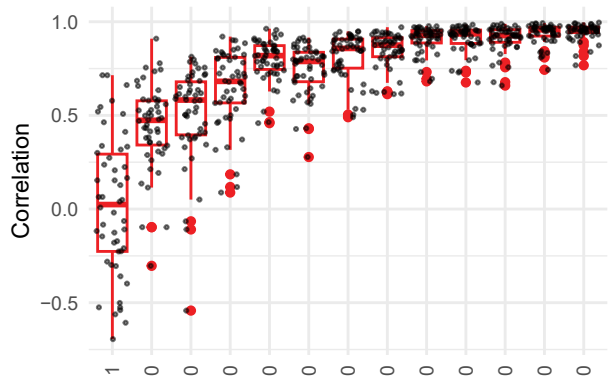**D**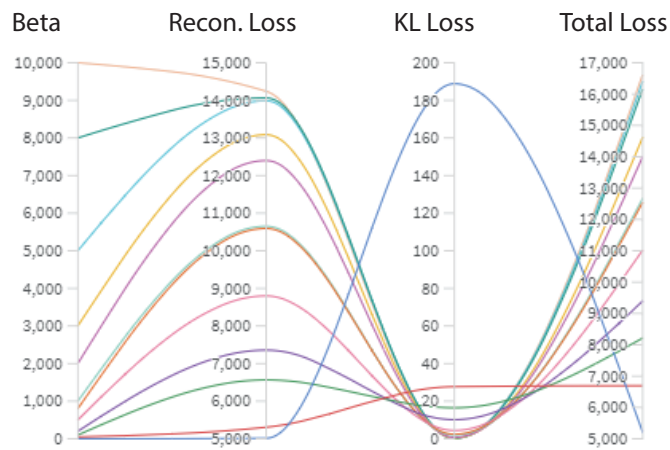**E**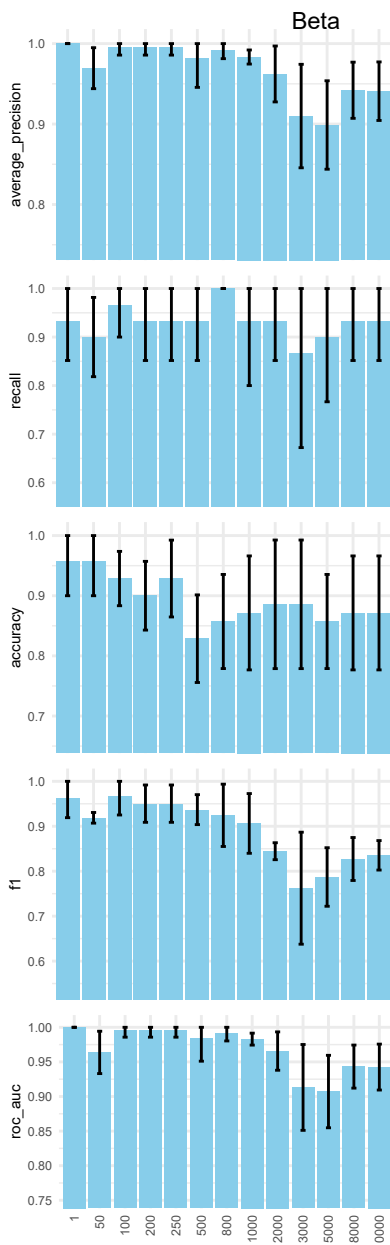**F**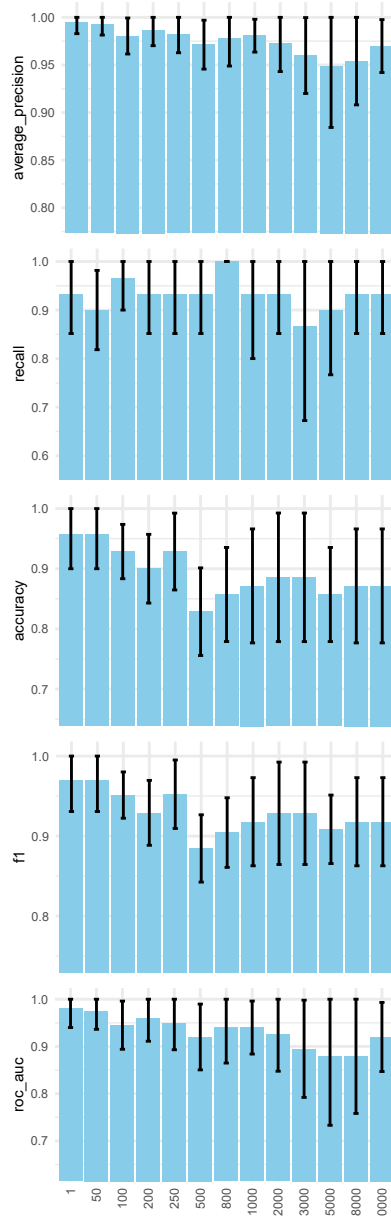**G**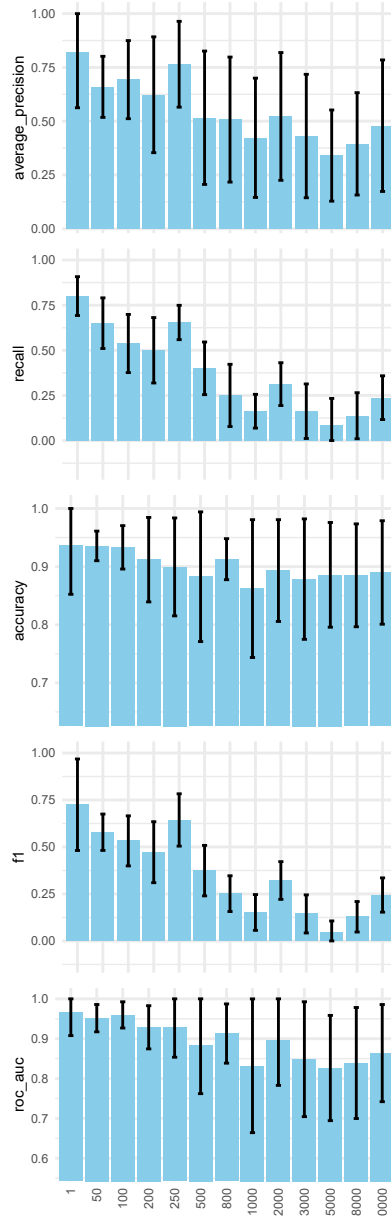

Supplement: S5 Fig — A sweep (random search) of the hyperparameter space was performed, resulting in 60 evaluated models. A: importance and correlation metrics provided by wandb. B: model results summarized by encoder architecture. X-axis labels refer to the size of the hidden layers in the encoder and decoder. C: Sweep results for the beta-priorVAE model across beta values, reporting the correlation between latent values and priors. D: Sweep results for beta, reconstruction loss, KL loss, and total loss. E-G: Effects of beta sweep on classification tasks for E: leukemia vs health, F: lung vs breast cancer, and G: tissue. (PDF) [file pcbi.1011198.s005.pdf]

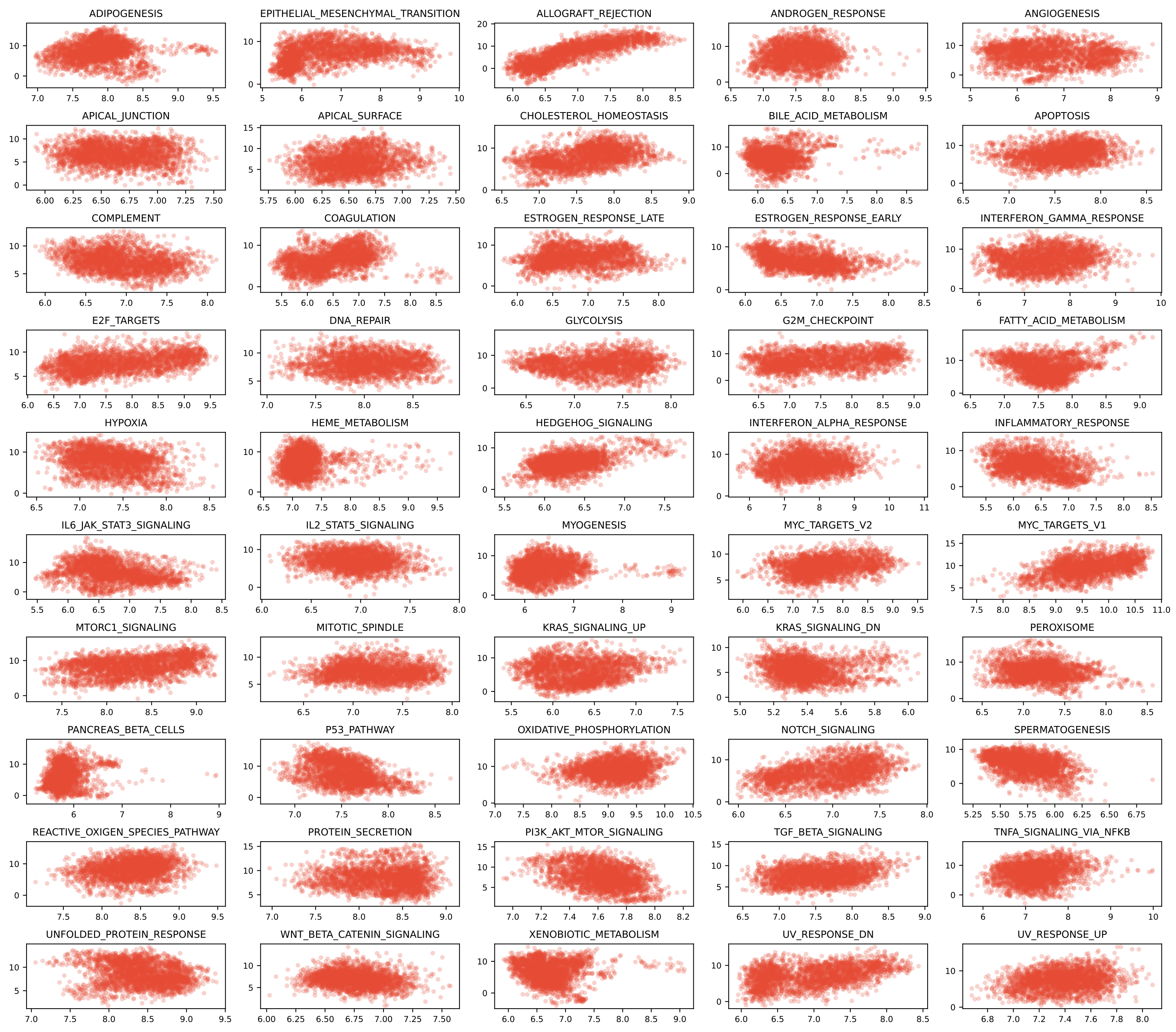

Supplement: S6 Fig — These plots show the extent to which the model adheres to the original meaning of the pathway labels. (ZIP) [file pcbi.1011198.s006.zip › S6A_Fig.pdf]

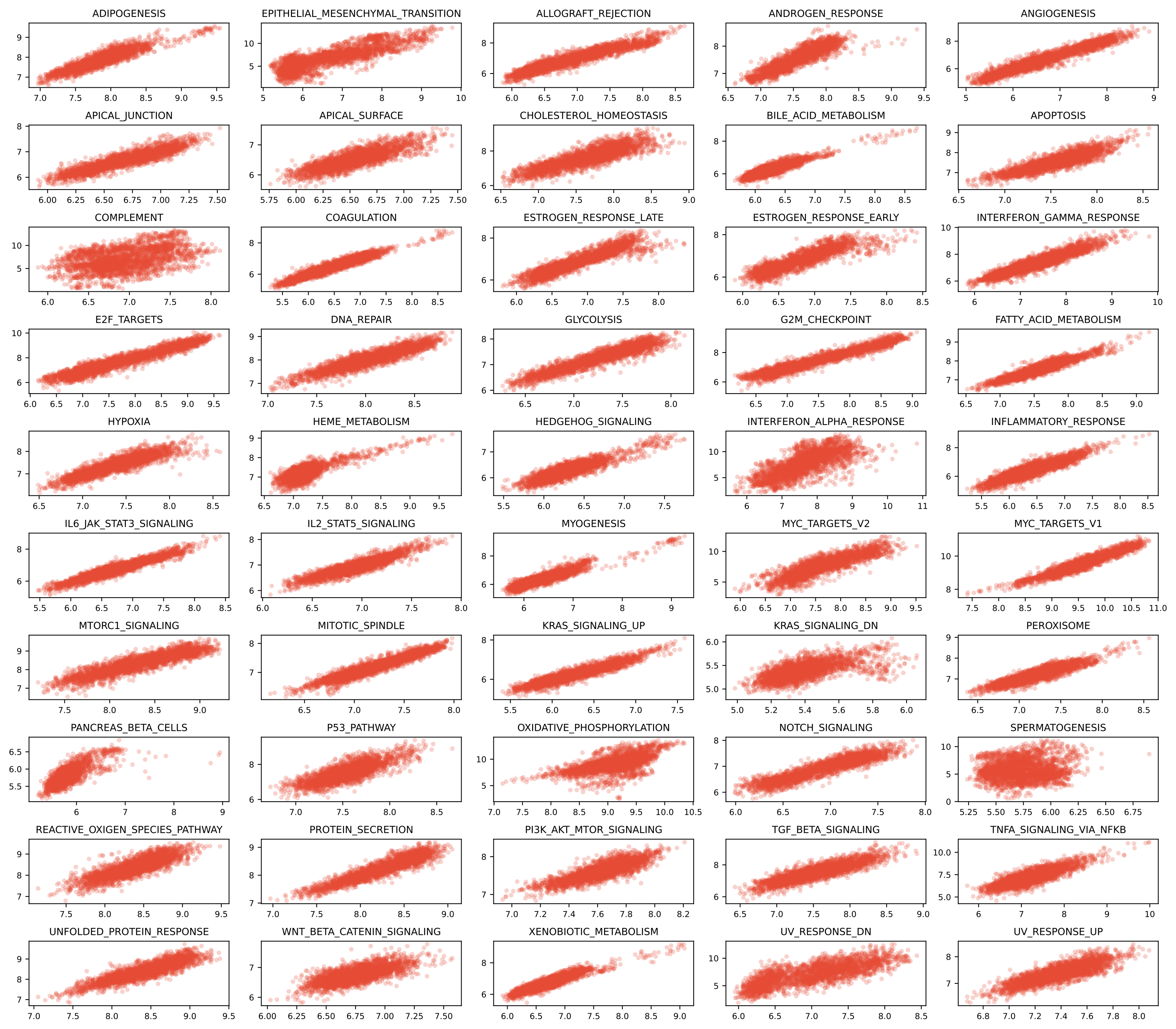

Supplement: S6 Fig — These plots show the extent to which the model adheres to the original meaning of the pathway labels. (ZIP) [file pcbi.1011198.s006.zip › S6B_Fig.pdf]

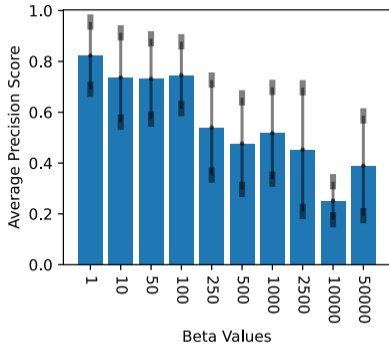

beta-simpleVAE

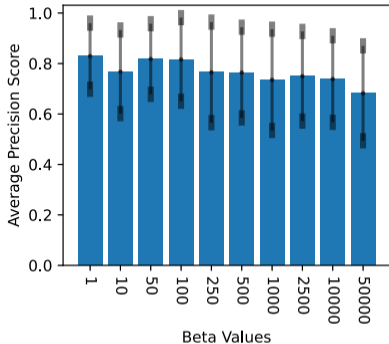

beta-priorVAE

Supplement: S10 Fig — (PDF) [file pcbi.1011198.s010.pdf]
