# Supplementary material for: A variational autoencoder trained with priors from canonical pathways increases the interpretability of transcriptome data
Source: PLoS Comput Biol. 2024 Jul 3;20(7):e1011198. doi: 10.1371/journal.pcbi.1011198 (PMC11251626; doi:10.1371/journal.pcbi.1011198)
Supplement: S8 Fig — Heatmaps show latent values pathways defined by A: MSigDB and B: KEGG. C and D show the top differentially expressed latent variables based on the p-value for MSigDB and KEGG respectively. (PDF) [file pcbi.1011198.s008.pdf]

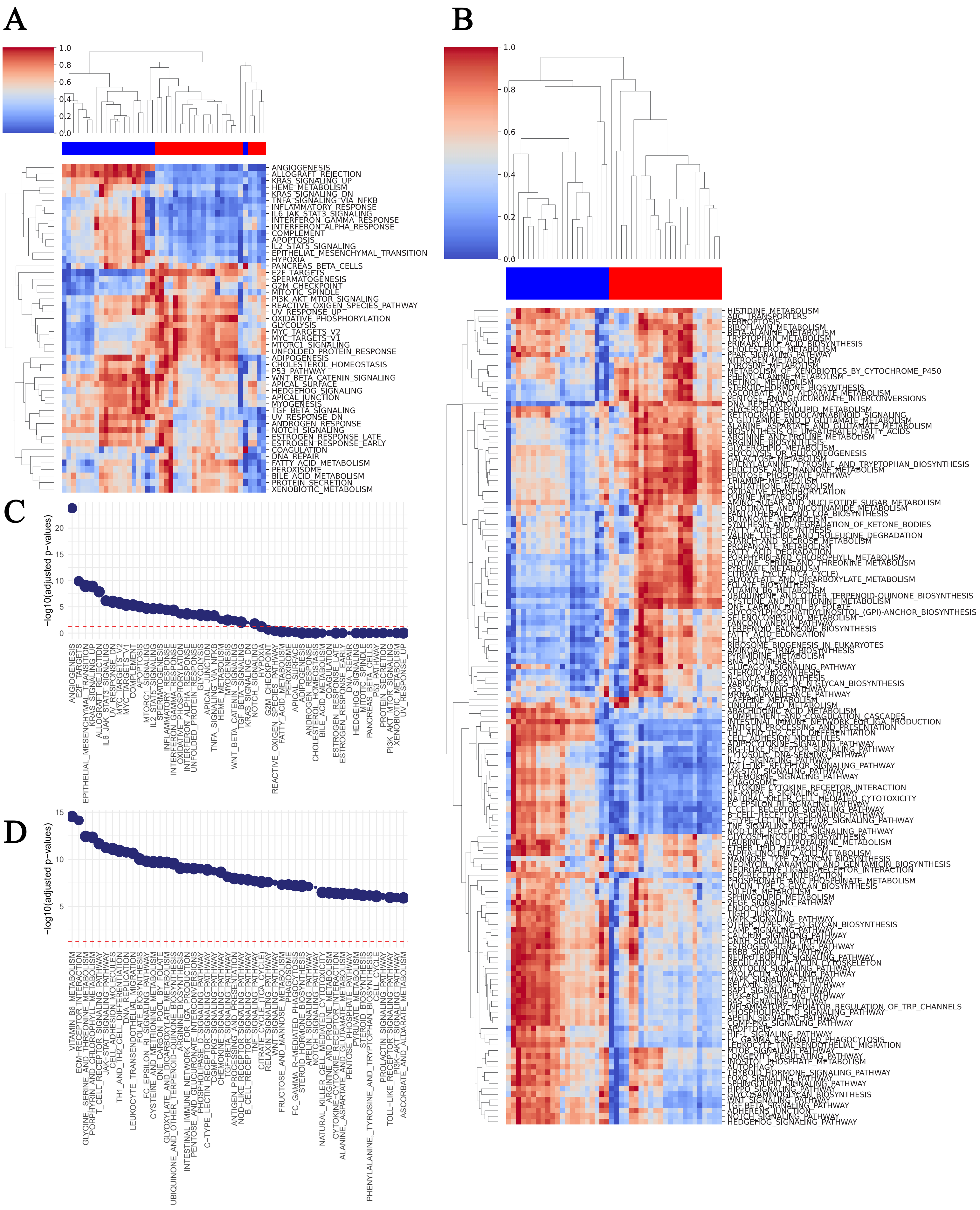

A
1.0
0.8
0.6
0.4
0.2
0.0
ANGIOGENESIS
ALLOGRAFT_REJECTION
KRAS_SIGNALING_UP
HEME_METABOLISM
KRAS_SIGNALING_DN
TNFA_SIGNALING_VIA_NFKB
INFLAMMATORY_RESPONSE
IL6_JAK_STAT3_SIGNALING
INTERFERON_GAMMA_RESPONSE
INTERFERON_ALPHA_RESPONSE
COMPLEMENT
APOPTOSIS
IL2_STAT5_SIGNALING
EPITHELIAL_MESENCHYMAL_TRANSITION
HYPOXIA
PANCREAS_BETA_CELLS
E2F_TARGETS
SPERMATOGENESIS
G2M_CHECKPOINT
MITOTIC_SPINDLE
PI3K_AKT_MTOR_SIGNALING
REACTIVE_OXIGEN_SPECIES_PATHWAY
UV_RESPONSE_UP
OXIDATIVE_PHOSPHORYLATION
GLYCOLYSIS
MYC_TARGETS_V2
MYC_TARGETS_V1
MTORC1_SIGNALING
UNFOLDED_PROTEIN_RESPONSE
ADIPOGENESIS
CHOLESTEROL_HOMEOSTASIS
P53_PATHWAY
WNT_BETA_CATENIN_SIGNALING
APICAL_SURFACE
HEDGEHOG_SIGNALING
APICAL_JUNCTION
MYOGENESIS
TGF_BETA_SIGNALING
UV_RESPONSE_DN
ANDROGEN_RESPONSE
NOTCH_SIGNALING
ESTROGEN_RESPONSE_LATE
ESTROGEN_RESPONSE_EARLY
COAGULATION
DNA_REPAIR
FATTY_ACID_METABOLISM
PEROXISOME
BILE_ACID_METABOLISM
PROTEIN_SECRETION
XENOBIOTIC_METABOLISM
B
1.0
0.8
0.6
0.4
0.2
0.0
HISTIDINE_METABOLISM
ABC_TRANSPORTERS
FERROPTOSIS
RIBOFLAVIN_METABOLISM
BETA-ALANINE_METABOLISM
TRYPTOPHAN_METABOLISM
PRIMARY_BILE_ACID_BIOSYNTHESIS
CHOLESTEROL_METABOLISM
PPAR_SIGNALING_PATHWAY
NITROGEN_METABOLISM
TYROSINE_METABOLISM
METABOLISM_OF_XENOBIOTICS_BY_CYTOCHROME_P450
PHENYLALANINE_METABOLISM
RETINOL_METABOLISM
STEROID_HORMONE_BIOSYNTHESIS
ASCORBATE_AND_ALDARATE_METABOLISM
PENTOSE_AND_GLUCURONATE_INTERCONVERSIONS
DNA_REPLICATION
GLYCEROPHOSPHOLIPID_METABOLISM
RETROGRADE_ENDOCANNABINOID_SIGNALING
D-GLUTAMINE_AND_D-GLUTAMATE_METABOLISM
ALANINE_ASPARTATE_AND_GLUTAMATE_METABOLISM
BIOSYNTHESIS_OF_UNSATURATED_FATTY_ACIDS
ARGININE_AND_PROLINE_METABOLISM
ARGININE_BIOSYNTHESIS
GLYCEROLIPID_METABOLISM
GLYCOLYSIS_OR_GLUCONEOGENESIS
GALACTOSE_METABOLISM
PHENYLALANINE_TYROSINE_AND_TRYPTOPHAN_BIOSYNTHESIS
FRUCTOSE_AND_MANNOSE_METABOLISM
PENTOSE_PHOSPHATE_PATHWAY
THIAMINE_METABOLISM
GLUTATHIONE_METABOLISM
OXIDATIVE_PHOSPHORYLATION
PURINE_METABOLISM
AMINO_SUGAR_AND_NUCLEOTIDE_SUGAR_METABOLISM
NICOTINATE_AND_NICOTINAMIDE_METABOLISM
PANTOTHENATE_AND_COA_BIOSYNTHESIS
BUTANOATE_METABOLISM
SYNTHESIS_AND_DEGRADATION_OF_KETONE_BODIES
FATTY_ACID_BIOSYNTHESIS
VALINE_LEUCINE_AND_ISOLEUCINE_DEGRADATION
STARCH_AND_SUCROSE_METABOLISM
PROPANOATE_METABOLISM
FATTY_ACID_DEGRADATION
PORPHYRIN_AND_CHLOROPHYLL_METABOLISM
GLYCINE_SERINE_AND_THREONINE_METABOLISM
PYRUVATE_METABOLISM
CITRATE_CYCLE_(TCA_CYCLE)
GLYOXYLATE_AND_DICARBOXYLATE_METABOLISM
VITAMIN_B6_METABOLISM
UBIQUINONE_AND_OTHER_TERPENOID-QUINONE_BIOSYNTHESIS
CYSTEINE_AND_METHIONINE_METABOLISM
ONE_CARBON_POOL_BY_FOLATE
GLYCOSYLPHOSPHATIDYLINOSITOL_(GPI)-ANCHOR_BIOSYNTHESIS
SELENOCOMPOUND_METABOLISM
FANCONI_ANEMIA_PATHWAY
TERPENOID_BACKBONE_BIOSYNTHESIS
FATTY_ACID_ELONGATION
CELL_CYCLE
RIBOSOME_BIOGENESIS_IN_EUKARYOTES
AMINOACYL-TRNA_BIOSYNTHESIS
PYRIMIDINE_METABOLISM
RNA_POLYMERASE
GLUCAGON_SIGNALING_PATHWAY
STEROID_BIOSYNTHESIS
N-GLYCAN_BIOSYNTHESIS
VARIOUS_TYPES_OF_N-GLYCAN_BIOSYNTHESIS
P53_SIGNALING_PATHWAY
MRNA_SURVEILLANCE_PATHWAY
CAFFEINE_METABOLISM
LINOLEIC_ACID_METABOLISM
ARACHIDONIC_ACID_METABOLISM
COMPLEMENT_AND_COAGULATION_CASCADES
INTESTINAL_IMMUNE_NETWORK_FOR_IGA_PRODUCTION
ANTIGEN_PROCESSING_AND_PRESENTATION
TH1_AND_TH2_CELL_DIFFERENTIATION
CELL_ADHESION_MOLECULES
ADIPOCYTOKINE_SIGNALING_PATHWAY
RIG-I-LIKE_RECEPTOR_SIGNALING_PATHWAY
CYTOSOLIC_DNA-SENSING_PATHWAY
IL-17_SIGNALING_PATHWAY
TOLL-LIKE_RECEPTOR_SIGNALING_PATHWAY
JAK-STAT_SIGNALING_PATHWAY
CHEMOKINE_SIGNALING_PATHWAY
PHAGOSOME
CYTOKINE-CYTOKINE_RECEPTOR_INTERACTION
NF-KAPPA_B_SIGNALING_PATHWAY
NATURAL_KILLER_CELL_MEDIATED_CYTOTOXICITY
FC_EPSILON_RI_SIGNALING_PATHWAY
T_CELL_RECEPTOR_SIGNALING_PATHWAY
B_CELL_RECEPTOR_SIGNALING_PATHWAY
C-TYPE_LECTIN_RECEPTOR_SIGNALING_PATHWAY
TNF_SIGNALING_PATHWAY
NOD-LIKE_RECEPTOR_SIGNALING_PATHWAY
GLYCOSPHINGOLIPID_BIOSYNTHESIS
TAURINE_AND_HYPOTAURINE_METABOLISM
ETHER_LIPID_METABOLISM
ALPHA-LINOLENIC_ACID_METABOLISM
MANNOSE_TYPE_O-GLYCAN_BIOSYNTHESIS
NEOMYCIN_KANAMYCIN_AND_GENTAMICIN_BIOSYNTHESIS
NEUROACTIVE_LIGAND_RECEPTOR_INTERACTION
ECM-RECEPTOR_INTERACTION
PHOSPHONATE_AND_PHOSPHINATE_METABOLISM
MUCIN_TYPE_O-GLYCAN_BIOSYNTHESIS
SULFUR_METABOLISM
SPHINGOLIPID_METABOLISM
VEGF_SIGNALING_PATHWAY
ENDOCYTOSIS
TIGHT_JUNCTION
AMPK_SIGNALING_PATHWAY
OTHER_TYPES_OF_O-GLYCAN_BIOSYNTHESIS
CAMP_SIGNALING_PATHWAY
CALCIUM_SIGNALING_PATHWAY
GNRH_SIGNALING_PATHWAY
ESTROGEN_SIGNALING_PATHWAY
ERBB_SIGNALING_PATHWAY
NEUROTROPHIN_SIGNALING_PATHWAY
REGULATION_OF_ACTIN_CYTOSKELETON
OXYTOCIN_SIGNALING_PATHWAY
PROLACTIN_SIGNALING_PATHWAY
MAPK_SIGNALING_PATHWAY
RELAXIN_SIGNALING_PATHWAY
RAP1_SIGNALING_PATHWAY
PI3K-AKT_SIGNALING_PATHWAY
RAS_SIGNALING_PATHWAY
INFLAMMATORY_MEDIATOR_REGULATION_OF_TRP_CHANNELS
PHOSPHOLIPASE_D_SIGNALING_PATHWAY
APELIN_SIGNALING_PATHWAY
CGMP-PKG_SIGNALING_PATHWAY
APOPTOSIS
HIF-1_SIGNALING_PATHWAY
FC_GAMMA_R-MEDIATED_PHAGOCYTOSIS
LEUKOCYTE_TRANSENDOTHELIAL_MIGRATION
MTOR_SIGNALING_PATHWAY
LONGEVITY_REGULATING_PATHWAY
INOSITOL_PHOSPHATE_METABOLISM
AUTOPHAGY
THYROID_HORMONE_SIGNALING_PATHWAY
FOXO_SIGNALING_PATHWAY
SPHINGOLIPID_SIGNALING_PATHWAY
HIPPO_SIGNALING_PATHWAY
GLYCOSAMINOGLYCAN_BIOSYNTHESIS
WNT_SIGNALING_PATHWAY
TGF-BETA_SIGNALING_PATHWAY
ADHERENS_JUNCTION
NOTCH_SIGNALING_PATHWAY
HEDGEHOG_SIGNALING_PATHWAY
C
-log10(adjusted p-values)
0
5
10
15
20
ANGIOGENESIS
E2F_TARGETS
EPITHELIAL_MESENCHYMAL_TRANSITION
KRAS_SIGNALING_UP
ALLOGRAFT_REJECTION
IL6_JAK_STAT3_SIGNALING
UV_RESPONSE_DN
MYC_TARGETS_V2
MYC_TARGETS_V1
COMPLEMENT
APOPTOSIS
MTORC1_SIGNALING
IL2_STAT5_SIGNALING
SPERMATOGENESIS
INFLAMMATORY_RESPONSE
INTERFERON_GAMMA_RESPONSE
OXIDATIVE_PHOSPHORYLATION
INTERFERON_ALPHA_RESPONSE
UNFOLDED_PROTEIN_RESPONSE
GLYCOLYSIS
APICAL_JUNCTION
TNFA_SIGNALING_VIA_NFKB
HEME_METABOLISM
MYOGENESIS
WNT_BETA_CATENIN_SIGNALING
TGF_BETA_SIGNALING
KRAS_SIGNALING_DN
NOTCH_SIGNALING
HYPOXIA
G2M_CHECKPOINT
REACTIVE_OXIGEN_SPECIES_PATHWAY
FATTY_ACID_METABOLISM
PEROXISOME
APICAL_SURFACE
ADIPOGENESIS
ANDROGEN_RESPONSE
CHOLESTEROL_HOMEOSTASIS
BILE_ACID_METABOLISM
COAGULATION
ESTROGEN_RESPONSE_LATE
ESTROGEN_RESPONSE_EARLY
DNA_REPAIR
HEDGEHOG_SIGNALING
MITOTIC_SPINDLE
PANCREAS_BETA_CELLS
P53_PATHWAY
PROTEIN_SECRETION
PI3K_AKT_MTOR_SIGNALING
XENOBIOTIC_METABOLISM
UV_RESPONSE_UP
D
-log10(adjusted p-values)
5
10
15
VITAMIN_B6_METABOLISM
GLYCINE_SERINE_AND_THREONINE_METABOLISM
ECM-RECEPTOR_INTERACTION
PORPHYRIN_AND_CHLOROPHYLL_METABOLISM
T_CELL_RECEPTOR_SIGNALING_PATHWAY
JAK-STAT_SIGNALING_PATHWAY
CELL_ADHESION_MOLECULES
TH1_AND_TH2_CELL_DIFFERENTIATION
DNA_REPLICATION
LEUKOCYTE_TRANSENDOTHELIAL_MIGRATION
FOLATE_BIOSYNTHESIS
FC_EPSILON_RI_SIGNALING_PATHWAY
CYSTEINE_AND_METHIONINE_METABOLISM
ONE_CARBON_POOL_BY_FOLATE
GLYOXYLATE_AND_DICARBOXYLATE_METABOLISM
UBIQUINONE_AND_OTHER_TERPENOID-QUINONE_BIOSYNTHESIS
ARGININE_BIOSYNTHESIS
INTESTINAL_IMMUNE_NETWORK_FOR_IGA_PRODUCTION
PENTOSE_AND_GLUCURONATE_INTERCONVERSIONS
PHOSPHOLIPASE_D_SIGNALING_PATHWAY
C-TYPE_LECTIN_RECEPTOR_SIGNALING_PATHWAY
CHEMOKINE_SIGNALING_PATHWAY
TGF-BETA_SIGNALING_PATHWAY
ANTIGEN_PROCESSING_AND_PRESENTATION
NOD-LIKE_RECEPTOR_SIGNALING_PATHWAY
B_CELL_RECEPTOR_SIGNALING_PATHWAY
TNF_SIGNALING_PATHWAY
CITRATE_CYCLE_(TCA_CYCLE)
RELAXIN_SIGNALING_PATHWAY
WNT_SIGNALING_PATHWAY
FRUCTOSE_AND_MANNOSE_METABOLISM
PHAGOSOME
FC_GAMMA_R-MEDIATED_PHAGOCYTOSIS
STEROID_HORMONE_BIOSYNTHESIS
APELIN_SIGNALING_PATHWAY
NOTCH_SIGNALING_PATHWAY
NATURAL_KILLER_CELL_MEDIATED_CYTOTOXICITY
ARGININE_AND_PROLINE_METABOLISM
CYTOKINE-CYTOKINE_RECEPTOR_INTERACTION
ALANINE_ASPARTATE_AND_GLUTAMATE_METABOLISM
PENTOSE_PHOSPHATE_PATHWAY
PYRUVATE_METABOLISM
STEROID_BIOSYNTHESIS
PHENYLALANINE_TYROSINE_AND_TRYPTOPHAN_BIOSYNTHESIS
CELL_CYCLE
PROLACTIN_SIGNALING_PATHWAY
TOLL-LIKE_RECEPTOR_SIGNALING_PATHWAY
PI3K-AKT_SIGNALING_PATHWAY
ASCORBATE_AND_ALDARATE_METABOLISM
