# Supplementary material for: A variational autoencoder trained with priors from canonical pathways increases the interpretability of transcriptome data
Source: PLoS Comput Biol. 2024 Jul 3;20(7):e1011198. doi: 10.1371/journal.pcbi.1011198 (PMC11251626; doi:10.1371/journal.pcbi.1011198)
Supplement: S7 Fig — Heatmaps show latent values pathways defined by A: MSigDB and B: KEGG. C and D show the top differentially expressed latent variables based on the p-value for MSigDB and KEGG respectively. (PDF) [file pcbi.1011198.s007.pdf]

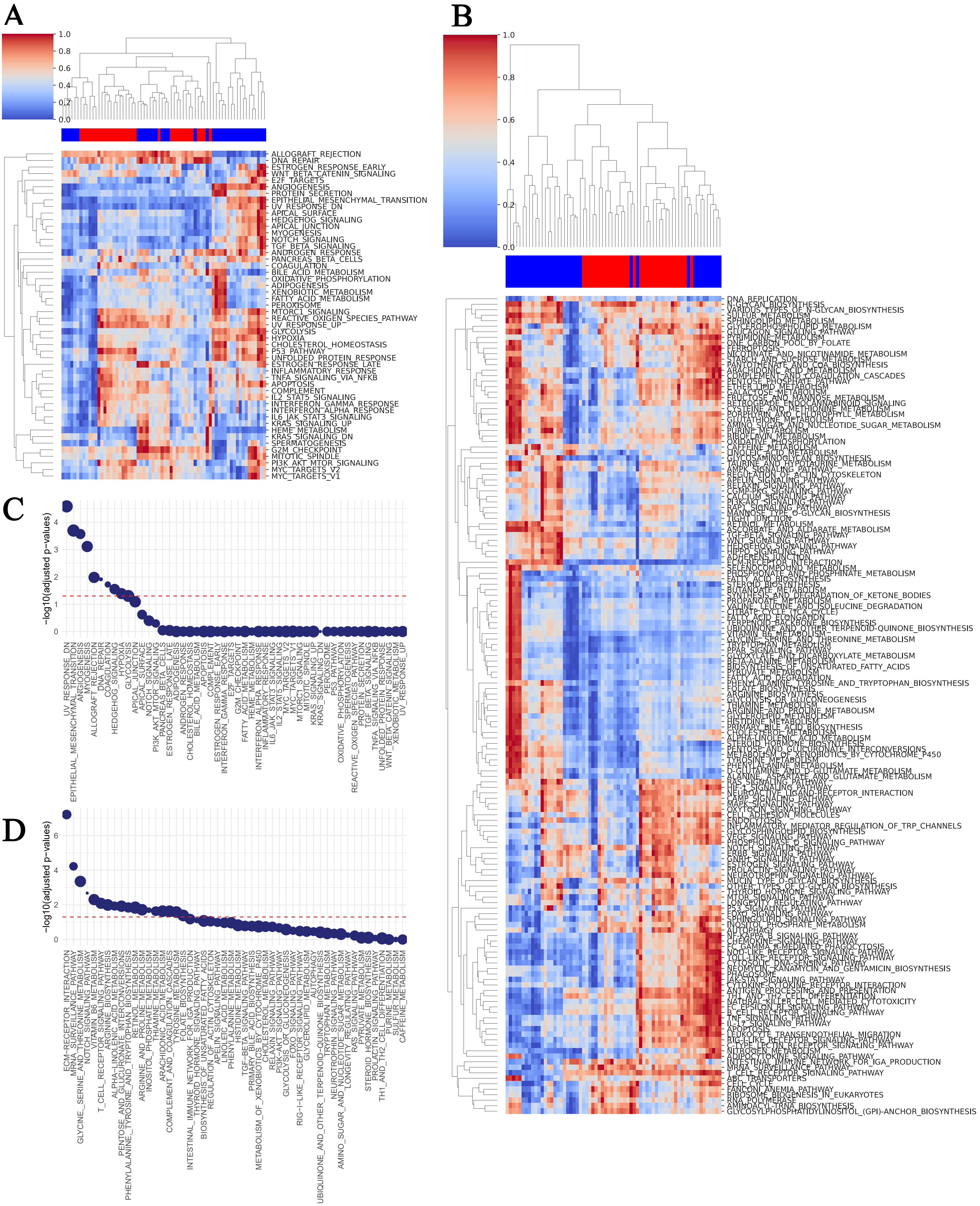
A
1.0
0.8
0.6
0.4
0.2
0.0
ALLOGRAFT_REJECTION
DNA_REPAIR
ESTROGEN_RESPONSE_EARLY
WNT_BETA_CATENIN_SIGNALING
E2F_TARGETS
ANGIOGENESIS
PROTEIN_SECRETION
EPITHELIAL_MESENCHYMAL_TRANSITION
UV_RESPONSE_DN
APICAL_SURFACE
HEDGEHOG_SIGNALING
APICAL_JUNCTION
MYOGENESIS
NOTCH_SIGNALING
TGF_BETA_SIGNALING
ANDROGEN_RESPONSE
PANCREAS_BETA_CELLS
COAGULATION
BILE_ACID_METABOLISM
OXIDATIVE_PHOSPHORYLATION
ADIPOGENESIS
XENOBIOTIC_METABOLISM
FATTY_ACID_METABOLISM
PEROXISOME
MTORC1_SIGNALING
REACTIVE_OXIGEN_SPECIES_PATHWAY
UV_RESPONSE_UP
GLYCOLYSIS
HYPOXIA
CHOLESTEROL_HOMEOSTASIS
P53_PATHWAY
UNFOLDED_PROTEIN_RESPONSE
ESTROGEN_RESPONSE_LATE
INFLAMMATORY_RESPONSE
TNFA_SIGNALING_VIA_NFKB
APOPTOSIS
COMPLEMENT
IL2_STAT5_SIGNALING
INTERFERON_GAMMA_RESPONSE
INTERFERON_ALPHA_RESPONSE
IL6_JAK_STAT3_SIGNALING
KRAS_SIGNALING_UP
HEME_METABOLISM
KRAS_SIGNALING_DN
SPERMATOGENESIS
G2M_CHECKPOINT
MITOTIC_SPINDLE
PI3K_AKT_MTOR_SIGNALING
MYC_TARGETS_V2
MYC_TARGETS_V1
B
1.0
0.8
0.6
0.4
0.2
0.0
DNA_REPLICATION
N-GLYCAN_BIOSYNTHESIS
VARIOUS_TYPES_OF_N-GLYCAN_BIOSYNTHESIS
SULFUR_METABOLISM
SPHINGOLIPID_METABOLISM
GLYCEROPHOSPHOLIPID_METABOLISM
GLUCAGON_SIGNALING_PATHWAY
PYRIMIDINE_METABOLISM
ONE_CARBON_POOL_BY_FOLATE
FERROPTOSIS
NICOTINATE_AND_NICOTINAMIDE_METABOLISM
STARCH_AND_SUCROSE_METABOLISM
PANTOTHENATE_AND_COA_BIOSYNTHESIS
ARACHIDONIC_ACID_METABOLISM
COMPLEMENT_AND_COAGULATION_CASCADES
PENTOSE_PHOSPHATE_PATHWAY
ETHER_LIPID_METABOLISM
GALACTOSE_METABOLISM
FRUCTOSE_AND_MANNOSE_METABOLISM
RETROGRADE_ENDOCANNABINOID_SIGNALING
CYSTEINE_AND_METHIONINE_METABOLISM
PORPHYRIN_AND_CHLOROPHYLL_METABOLISM
GLUTATHIONE_METABOLISM
AMINO_SUGAR_AND_NUCLEOTIDE_SUGAR_METABOLISM
PURINE_METABOLISM
RIBOFLAVIN_METABOLISM
OXIDATIVE_PHOSPHORYLATION
CAFFEINE_METABOLISM
LINOLEIC_ACID_METABOLISM
GLYCOSAMINOGLYCAN_BIOSYNTHESIS
TAURINE_AND_HYPOTAURINE_METABOLISM
AMPK_SIGNALING_PATHWAY
REGULATION_OF_ACTIN_CYTOSKELETON
APELIN_SIGNALING_PATHWAY
RELAXIN_SIGNALING_PATHWAY
CGMP-PKG_SIGNALING_PATHWAY
CALCIUM_SIGNALING_PATHWAY
PI3K-AKT_SIGNALING_PATHWAY
RAP1_SIGNALING_PATHWAY
MANNOSE_TYPE_O-GLYCAN_BIOSYNTHESIS
TIGHT_JUNCTION
RETINOL_METABOLISM
ASCORBATE_AND_ALDARATE_METABOLISM
TGF-BETA_SIGNALING_PATHWAY
WNT_SIGNALING_PATHWAY
HEDGEHOG_SIGNALING_PATHWAY
HIPPO_SIGNALING_PATHWAY
ADHERENS_JUNCTION
ECM-RECEPTOR_INTERACTION
SELENOCOMPOUND_METABOLISM
PHOSPHONATE_AND_PHOSPHINATE_METABOLISM
FATTY_ACID_BIOSYNTHESIS
STEROID_BIOSYNTHESIS
BUTANOATE_METABOLISM
SYNTHESIS_AND_DEGRADATION_OF_KETONE_BODIES
PROPANOATE_METABOLISM
VALINE,_LEUCINE_AND_ISOLEUCINE_DEGRADATION
CITRATE_CYCLE_(TCA_CYCLE)
FATTY_ACID_ELONGATION
TERPENOID_BACKBONE_BIOSYNTHESIS
UBIQUINONE_AND_OTHER_TERPENOID-QUINONE_BIOSYNTHESIS
VITAMIN_B6_METABOLISM
GLYCINE,_SERINE_AND_THREONINE_METABOLISM
TRYPTOPHAN_METABOLISM
PPAR_SIGNALING_PATHWAY
GLYOXYLATE_AND_DICARBOXYLATE_METABOLISM
BETA-ALANINE_METABOLISM
BIOSYNTHESIS_OF_UNSATURATED_FATTY_ACIDS
PYRUVATE_METABOLISM
FATTY_ACID_DEGRADATION
PHENYLALANINE,_TYROSINE_AND_TRYPTOPHAN_BIOSYNTHESIS
FOLATE_BIOSYNTHESIS
ARGININE_BIOSYNTHESIS
GLYCOLYSIS_OR_GLUCONEOGENESIS
THIAMINE_METABOLISM
ARGININE_AND_PROLINE_METABOLISM
GLYCEROLIPID_METABOLISM
HISTIDINE_METABOLISM
PRIMARY_BILE_ACID_BIOSYNTHESIS
CHOLESTEROL_METABOLISM
ALPHA-LINOLENIC_ACID_METABOLISM
STEROID_HORMONE_BIOSYNTHESIS
PENTOSE_AND_GLUCURONATE_INTERCONVERSIONS
METABOLISM_OF_XENOBIOTICS_BY_CYTOCHROME_P450
TYROSINE_METABOLISM
PHENYLALANINE_METABOLISM
D-GLUTAMINE_AND_D-GLUTAMATE_METABOLISM
ALANINE,_ASPARTATE_AND_GLUTAMATE_METABOLISM
RAS_SIGNALING_PATHWAY
HIF-1_SIGNALING_PATHWAY
NEUROACTIVE_LIGAND-RECEPTOR_INTERACTION
CAMP_SIGNALING_PATHWAY
MAPK_SIGNALING_PATHWAY
OXYTOCIN_SIGNALING_PATHWAY
CELL_ADHESION_MOLECULES
ENDOCYTOSIS
INFLAMMATORY_MEDIATOR_REGULATION_OF_TRP_CHANNELS
GLYCOSPHINGOLIPID_BIOSYNTHESIS
VEGF_SIGNALING_PATHWAY
PHOSPHOLIPASE_D_SIGNALING_PATHWAY
NOTCH_SIGNALING_PATHWAY
ERBB_SIGNALING_PATHWAY
GNRH_SIGNALING_PATHWAY
ESTROGEN_SIGNALING_PATHWAY
PROLACTIN_SIGNALING_PATHWAY
NEUROTROPHIN_SIGNALING_PATHWAY
MUCIN_TYPE_O-GLYCAN_BIOSYNTHESIS
OTHER_TYPES_OF_O-GLYCAN_BIOSYNTHESIS
THYROID_HORMONE_SIGNALING_PATHWAY
MTOR_SIGNALING_PATHWAY
LONGEVITY_REGULATING_PATHWAY
P53_SIGNALING_PATHWAY
FOXO_SIGNALING_PATHWAY
SPHINGOLIPID_SIGNALING_PATHWAY
INOSITOL_PHOSPHATE_METABOLISM
AUTOPHAGY
NF-KAPPA_B_SIGNALING_PATHWAY
CHEMOKINE_SIGNALING_PATHWAY
FC_GAMMA_R-MEDIATED_PHAGOCYTOSIS
NOD-LIKE_RECEPTOR_SIGNALING_PATHWAY
TOLL-LIKE_RECEPTOR_SIGNALING_PATHWAY
CYTOSOLIC_DNA-SENSING_PATHWAY
NEOMYCIN,_KANAMYCIN_AND_GENTAMICIN_BIOSYNTHESIS
PHAGOSOME
JAK-STAT_SIGNALING_PATHWAY
CYTOKINE-CYTOKINE_RECEPTOR_INTERACTION
ANTIGEN_PROCESSING_AND_PRESENTATION
TH1_AND_TH2_CELL_DIFFERENTIATION
NATURAL_KILLER_CELL_MEDIATED_CYTOTOXICITY
FC_EPSILON_RI_SIGNALING_PATHWAY
B_CELL_RECEPTOR_SIGNALING_PATHWAY
TNF_SIGNALING_PATHWAY
IL-17_SIGNALING_PATHWAY
APOPTOSIS
LEUKOCYTE_TRANSENDOTHELIAL_MIGRATION
RIG-I-LIKE_RECEPTOR_SIGNALING_PATHWAY
C-TYPE_LECTIN_RECEPTOR_SIGNALING_PATHWAY
NITROGEN_METABOLISM
ADIPOCYTOKINE_SIGNALING_PATHWAY
INTESTINAL_IMMUNE_NETWORK_FOR_IGA_PRODUCTION
MRNA_SURVEILLANCE_PATHWAY
T_CELL_RECEPTOR_SIGNALING_PATHWAY
ABC_TRANSPORTERS
CELL_CYCLE
FANCONI_ANEMIA_PATHWAY
RIBOSOME_BIOGENESIS_IN_EUKARYOTES
RNA_POLYMERASE
AMINOACYL-TRNA_BIOSYNTHESIS
GLYCOSYLPHOSPHATIDYLINOSITOL_(GPI)-ANCHOR_BIOSYNTHESIS
C
−log10(adjusted p−values)
0
1
2
3
4
UV_RESPONSE_DN
EPITHELIAL_MESENCHYMAL_TRANSITION
ANGIOGENESIS
MYOGENESIS
ALLOGRAFT_REJECTION
DNA_REPAIR
COAGULATION
HEDGEHOG_SIGNALING
HYPOXIA
GLYCOLYSIS
APICAL_JUNCTION
APICAL_SURFACE
NOTCH_SIGNALING
PI3K_AKT_MTOR_SIGNALING
PANCREAS_BETA_CELLS
ESTROGEN_RESPONSE_LATE
ADIPOGENESIS
ANDROGEN_RESPONSE
CHOLESTEROL_HOMEOSTASIS
BILE_ACID_METABOLISM
COMPLEMENT
ESTROGEN_RESPONSE_EARLY
INTERFERON_GAMMA_RESPONSE
E2F_TARGETS
G2M_CHECKPOINT
FATTY_ACID_METABOLISM
HEME_METABOLISM
INTERFERON_ALPHA_RESPONSE
INFLAMMATORY_RESPONSE
IL6_JAK_STAT3_SIGNALING
IL2_STAT5_SIGNALING
MYC_TARGETS_V2
MYC_TARGETS_V1
MTORC1_SIGNALING
MITOTIC_SPINDLE
KRAS_SIGNALING_DN
KRAS_SIGNALING_UP
PEROXISOME
P53_PATHWAY
OXIDATIVE_PHOSPHORYLATION
SPERMATOGENESIS
REACTIVE_OXIGEN_SPECIES_PATHWAY
PROTEIN_SECRETION
TGF_BETA_SIGNALING
TNFA_SIGNALING_VIA_NFKB
UNFOLDED_PROTEIN_RESPONSE
WNT_BETA_CATENIN_SIGNALING
XENOBIOTIC_METABOLISM
UV_RESPONSE_UP
D
−log10(adjusted p−values)
0
2
4
6
ECM-RECEPTOR_INTERACTION
MRNA_SURVEILLANCE_PATHWAY
GLYCINE,_SERINE_AND_THREONINE_METABOLISM
NOTCH_SIGNALING_PATHWAY
VITAMIN_B6_METABOLISM
T_CELL_RECEPTOR_SIGNALING_PATHWAY
ARGININE_BIOSYNTHESIS
ALPHA-LINOLENIC_ACID_METABOLISM
PENTOSE_AND_GLUCURONATE_INTERCONVERSIONS
PHENYLALANINE,_TYROSINE_AND_TRYPTOPHAN_BIOSYNTHESIS
RETINOL_METABOLISM
ARGININE_AND_PROLINE_METABOLISM
INOSITOL_PHOSPHATE_METABOLISM
THIAMINE_METABOLISM
ARACHIDONIC_ACID_METABOLISM
COMPLEMENT_AND_COAGULATION_CASCADES
TYROSINE_METABOLISM
FOLATE_BIOSYNTHESIS
INTESTINAL_IMMUNE_NETWORK_FOR_IGA_PRODUCTION
THYROID_HORMONE_SIGNALING_PATHWAY
BIOSYNTHESIS_OF_UNSATURATED_FATTY_ACIDS
REGULATION_OF_ACTIN_CYTOSKELETON
APELIN_SIGNALING_PATHWAY
LINOLEIC_ACID_METABOLISM
PHENYLALANINE_METABOLISM
HISTIDINE_METABOLISM
TGF-BETA_SIGNALING_PATHWAY
PRIMARY_BILE_ACID_BIOSYNTHESIS
METABOLISM_OF_XENOBIOTICS_BY_CYTOCHROME_P450
CHOLESTEROL_METABOLISM
RELAXIN_SIGNALING_PATHWAY
PI3K-AKT_SIGNALING_PATHWAY
GLYCOLYSIS_OR_GLUCONEOGENESIS
FOXO_SIGNALING_PATHWAY
RIG-I-LIKE_RECEPTOR_SIGNALING_PATHWAY
GLYCEROLIPID_METABOLISM
AUTOPHAGY
UBIQUINONE_AND_OTHER_TERPENOID-QUINONE_BIOSYNTHESIS
TRYPTOPHAN_METABOLISM
NEUROTROPHIN_SIGNALING_PATHWAY
AMINO_SUGAR_AND_NUCLEOTIDE_SUGAR_METABOLISM
LONGEVITY_REGULATING_PATHWAY
RAS_SIGNALING_PATHWAY
PPAR_SIGNALING_PATHWAY
STEROID_HORMONE_BIOSYNTHESIS
PROLACTIN_SIGNALING_PATHWAY
TH1_AND_TH2_CELL_DIFFERENTIATION
PURINE_METABOLISM
SULFUR_METABOLISM
CAFFEINE_METABOLISM
