# Supplementary material for: A variational autoencoder trained with priors from canonical pathways increases the interpretability of transcriptome data
Source: PLoS Comput Biol. 2024 Jul 3;20(7):e1011198. doi: 10.1371/journal.pcbi.1011198 (PMC11251626; doi:10.1371/journal.pcbi.1011198)
Supplement: S9 Fig — Heatmaps show latent values pathways defined by A: MSigDB and B: KEGG. C and D show the top differentially expressed latent variables based on the p-value for MSigDB and KEGG respectively. (PDF) [file pcbi.1011198.s009.pdf]

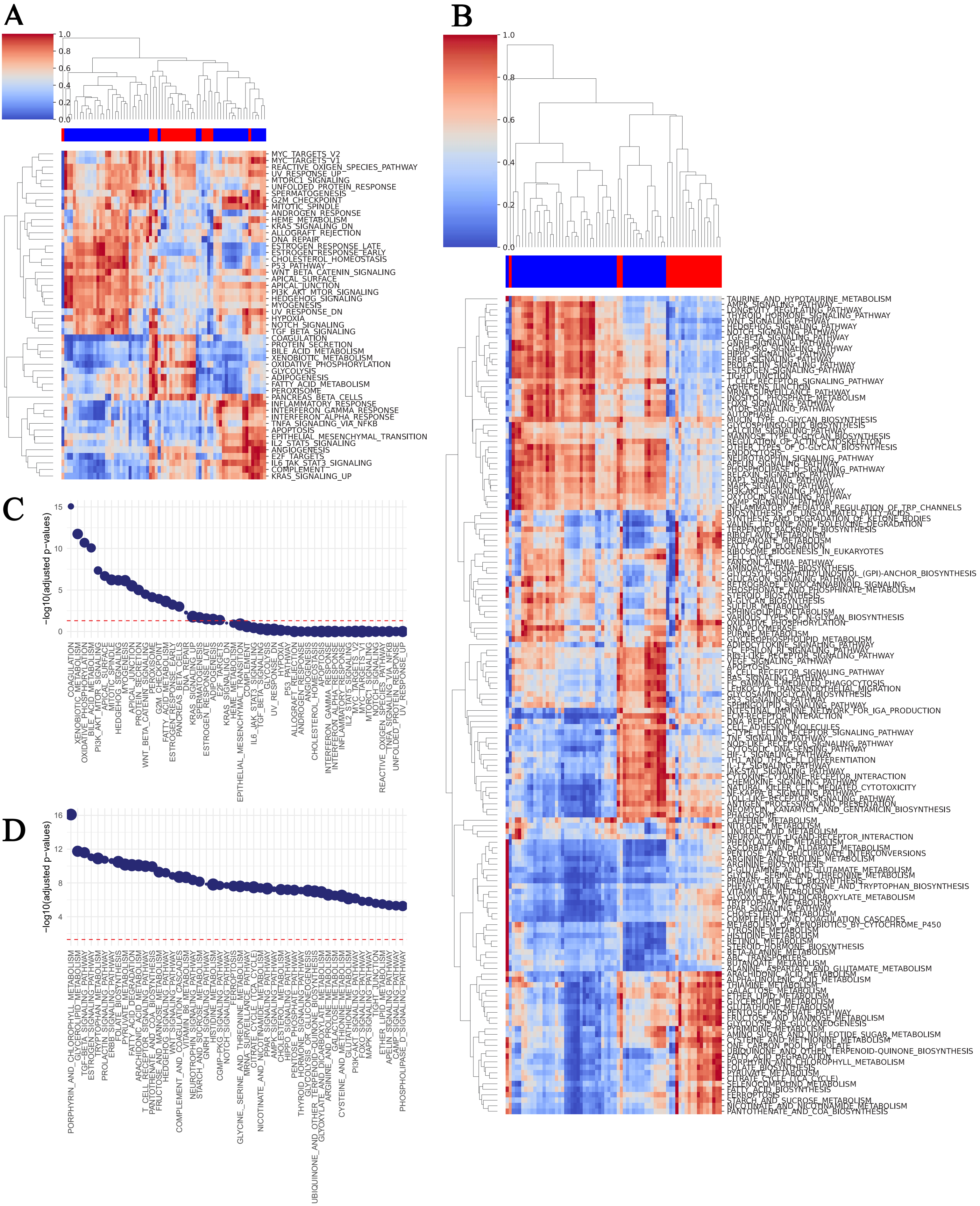
A
B
C
D
1.0
0.8
0.6
0.4
0.2
0.0
MYC_TARGETS_V2
MYC_TARGETS_V1
REACTIVE_OXIGEN_SPECIES_PATHWAY
UV_RESPONSE_UP
MTORC1_SIGNALING
UNFOLDED_PROTEIN_RESPONSE
SPERMATOGENESIS
G2M_CHECKPOINT
MITOTIC_SPINDLE
ANDROGEN_RESPONSE
HEME_METABOLISM
KRAS_SIGNALING_DN
ALLOGRAFT_REJECTION
DNA_REPAIR
ESTROGEN_RESPONSE_LATE
ESTROGEN_RESPONSE_EARLY
CHOLESTEROL_HOMEOSTASIS
P53_PATHWAY
WNT_BETA_CATENIN_SIGNALING
APICAL_SURFACE
APICAL_JUNCTION
PI3K_AKT_MTOR_SIGNALING
HEDGEHOG_SIGNALING
MYOGENESIS
UV_RESPONSE_DN
HYPOXIA
NOTCH_SIGNALING
TGF_BETA_SIGNALING
COAGULATION
PROTEIN_SECRETION
BILE_ACID_METABOLISM
XENOBIOTIC_METABOLISM
OXIDATIVE_PHOSPHORYLATION
GLYCOLYSIS
ADIPOGENESIS
FATTY_ACID_METABOLISM
PEROXISOME
PANCREAS_BETA_CELLS
INFLAMMATORY_RESPONSE
INTERFERON_GAMMA_RESPONSE
INTERFERON_ALPHA_RESPONSE
TNFA_SIGNALING_VIA_NFKB
APOPTOSIS
EPITHELIAL_MESENCHYMAL_TRANSITION
IL2_STAT5_SIGNALING
ANGIOGENESIS
E2F_TARGETS
IL6_JAK_STAT3_SIGNALING
COMPLEMENT
KRAS_SIGNALING_UP
−log10(adjusted p−values)
15
10
5
0
16
12
8
4
