# Supplementary material for: A variational autoencoder trained with priors from canonical pathways increases the interpretability of transcriptome data
Source: PLoS Comput Biol. 2024 Jul 3;20(7):e1011198. doi: 10.1371/journal.pcbi.1011198 (PMC11251626; doi:10.1371/journal.pcbi.1011198)
Supplement: Attachment — Submitted filename: Response the reviewers.pdf [file pcbi.1011198.s011.pdf]

Dear Editor, Dear Reviewers,

We would like to thank the reviewers for all their efforts examining our research contribution and providing valuable feedback and suggestions. We have made a herculean effort to address all of these suggestions and are very satisfied that the review process has greatly improved our paper.

Below we provide the original review comments and our responses to each issue one by one in blue.

Reviewer #1:

Here, the authors propose the use of autoencoder variations to increase interpretability of deep learning in transcriptomics data. More specifically, the novelty of this paper is the addition of canonical biological pathways as priors to the beta-variational autoencoder model. The authors reasoned that the ability to tune the prior weights with the beta hyperparameter ties the latent space to the curated biological pathways and henceforth providing better biological interpretability.

While the conceptual premise seems interesting, there are a few aspects I would like to see further explored:

1. - Table 2 provided showed that the introduction of beta drastically increases the reconstruction loss and reduces the KL divergence. However, the relationship between the beta hyperparameter and the two losses warrants more investigation – for example, how does the beta hyperparameter scale with respect to each loss? This may allow for a more fine-tuned approach to selecting the optimal amount of trade-off mentioned in the paper.

   Our Response: Thank you for your suggestion of having more exploration on the hyperparameter beta. We have added a results section (Results→Impact of hyperparameter choices on performance) evaluating the choices of hyperparamters in general, with particular emphasis on beta. Supplemental figure S5 reports the impact of the choice of beta on the correlations between latent and prior values, total loss, reconstruction loss, and KL divergence. Furthermore, we applied the biological classification benchmarks to the beta sweep as well and reported these results in the figure panel as well.

2. - The authors should expand on the classification metrics as well provide statistical tests to these metrics. Even though the authors attempted to justify the worse model accuracies with the poorer reconstruction losses, it remains unclear why the beta-prior models are underperforming compared to the simple

counterparts. This argues against the premise and warrants more investigation. Providing more classification metrics may provide an insight into why the models are worse off. This also ties into previous point, if the reconstruction loss scales accordingly to beta hyperparameter, it will be evident in the classification metrics.

Thank you for the suggestion, and we have expanded the classification metrics accordingly, as well as expanded the set of biological classification tasks to provide a clearer view of the trade off associated with the beta parameter. The metrics now include precision, recall, accuracy, f1 and AUC, and error bars are provided as well. Our biological benchmarks include Leukemia vs Normal, Lung vs Breast Cancer SC vs NSC Lung Cancer, and Tissue Classification. Please refer to the following figures which incorporate these additions: Figures, 3,4, S4.

3. Even though the argument for have biologically-relevant priors is logically sound, it requires more testing to determine if the effect is true. A good sanity check would be to introduce random biological sets and see if it reduces the model's classification performance. Another potential check would be to see if there are specific genes that are heavily influencing the models. For example, by only using subsets of the gene sets, one can test for the robustness of the biological priors.

   It is quite interesting to ask the question as to how much useful information is contained in the biological priors, and to what extent the model can take advantage of that information. The suggestion for randomizing the pathways to make a direct comparison possible is quite sensible. Taking this approach to its conclusion, the randomized pathways provide priors distributions that are essentially unit gaussians (centered around zero), which means that the model simply devolves into the one in our analysis we call "SimpleVAE" and "Beta-SimpleVAE". The direct comparison of the "Beta-SimpleVAE" and the "Beta-PriorVAE" shows that when we put a strong emphasis on the KL divergence term, then the biological information provided by the priors is essential to the performance of the model (Beta-PriorVAE consistently outperforms "Beta SimpleVAE"). This result does in a way prove there is real informational value in the pathway-derived priors. However, since the other non-prior-based models perform well across our benchmarks, our results also show that such models can learn the necessary features on their own, without the priors. Just to reiterate, we still find the prior-based model compelling because it gives us a link with human-understandable concepts to facilitate the interpretation of the results.

   Regarding an analysis of a subset of genes, this has been done as part of the community detection experiment. We have improved the presentation of evaluation of the community detection to make it more clear. There is indeed a performance hit associated with this reduction and this is now addressed in the discussion.

4. - While I agree that gene-level features are sufficient, I would like to see the effect of different normalization methods on the gene-level features. It is well known that different normalization methods can affect the downstream RNA-seq data analysis. As such, the authors should consider different normalization approaches.

   Thank you for mentioning normalization strategies. We had evaluated alternative normalization strategies previously and now include those results in the results section. Specifically, we were interested in whether relative changes in expression instead of absolute changes in expression would be more true to the biological impact of changes in expression. We observed that normalizing expression data with z-score transformation in fact decreased the performance.

   It is worth noting that our data set is based on the Affymetrix HG-U133Plus2 platform, which is microarray data and the RMA (Robust Multiarray Averaging) normalization strategy for microarray data is well established and perhaps not as much a subject of debate as is the case with RNA-seq. Looking forward to integrating diverse platform types, we certainly share the reviewer's view that assessing the impact of scales and normalization will be of great importance.

5. - While the premise of the paper is to prove the utility of biologically-informed priors, it might be useful to see if different pathway definitions from different databases may result in different results.

   Yes, thank you for this suggestion, as we were also very interested in exploring alternative pathway databases. We have now implemented the model using KEGG pathways in addition to the original MSigDB hallmarks. KEGG gives a different view on the transcriptome, heavy on metabolic pathways for example. There was notable consensus with the MSigDB model for the comparison of the two types of lung cancers, but also discrepancies for some of the other experiments. The tissue benchmark shows that KEGG plus a wild card dimension performed best at the tissue classification task. Please refer to updated figures 4, 6, S7, S8, and S9.

6. - The scope of disease classification tasks should be expanded to include other types of cancers and disease to test the statistical robustness of the models. Different physiological conditions manifest into different statistical properties of the data distributions observed. As such, it will be prudent to find out what type of statistical distribution causes the model to perform better or worse.

   Thank you for the suggestion. In the revised manuscript, we apply five validation tasks: (i) distinguishing leukemia vs normal bone marrow, (ii) distinguishing lung vs breast adenocarcinoma, (iii) lung adenocarcinoma vs healthy lung, (iv) lung adenocarcinoma vs small cell lung cancer, and (v) distinguishing tissue types. Of these five tasks, two were too trivial to provide a proper point of comparison among the models: lung adenocarcinoma vs healthy, and lung adenocarcinoma

vs small cell lung cancer. However, these comparisons could still be used later in our evaluation of differential pathway analysis.

7. - A good sanity check would be to see if the same pathways are derived when doing conventional differential gene expression analysis with the same classification groups. This may provide an insight into the advantages of using a machine-learning approach shown in this paper.

   Thank you for the suggestion, we have added the DEG results either from limma (corrected for batch effect) their corresponding EnricR results. In short we found the traditional method to produce a mix of reasonable results but also some sensitivity to RNA quality artifacts. Please refer for to Table 3 in the revised manuscript.

Reviewer #2:

The authors proposed autoencoders to compute latent representation for transcriptome signals. Five autoencorders, such as simpleAE, simpleVAE, Beta-SimpleVAE, PriorVAE, and Beta-PriorVAE, were compared for the assessment. The problem in the manuscript is interesting, but there are several major concerns before the publication.

1. - The approaches need to be clarified. The data processing and pipeline for the transcript level input, gene level input, and community level input are not clear. I strongly encourage the authors to improve the method.

   Thank you for this feedback. We have worked over the methods section accordingly, providing also more context, motivation, and justification for the steps involved. Upon reflection of this comment and the ones to follow, we think that we have identified Figure 1 as a source of confusion because it seemed like all 3 input types were part of the same model run. We have revised the figure, as well as the legend and some areas in the manuscript so that readers will not be misled.

2. - What's definition of community level input? Why is important? And for the community detection, why is KNN-based graph the best solution? Why not network inference methods?

   The original data are provided in the form of transcript expression levels. Transcripts can be summarized into genes, and from there we attempt to simplify the data further (a kind of feature selection) by using communities of genes. The idea of a community is that it is a collection of genes whose expression levels are highly correlated, and therefore it would be reasonable to select a single gene as

a representative for each community and greatly reduce the number of genes being considered for input into our model.

It is important to reduce the size of the input layer because this will also greatly reduce the number of parameters in the neural network that will have to be optimized. The hope and expectation is that the reduction will make the network less prone to overfitting and more likely to find a latent representation that is a good abstraction of the original data.

We cannot claim that the KNN-Leiden community detection strategy that we implemented is the best solution. It certainly seems like quite a reasonable solution and has a lot in common with other network inference methods. The community-based approach did produce better results in our hands compared to transcript-level input, but we did not see a tangible improvement over the gene-level input. We cannot exclude the possibility that further improvement to the community detection step could in fact make a meaningful improvement in our transcriptome modeling, and perhaps there are network inference algorithms that others have developed that would do just that.

Based on questions provided by the reviewer, and subsequent reflection by the authors, we have updated the Methods section for clarity, and added a new paragraph to the Discussion section to address these topics. We have also provided a new supplemental figure which makes the results much easier to interpret: Supplemental Figure S1 (particularly the box plots).

3. - Autoencoder is trained on each pathway or with whole gene level?

   This is an important question, and I can see where the confusion comes from. The input layer of the neural network consists of gene-level expression values. The trick with our system is that the information about the pathways involved is represented in the middle layer of the neural network, and although we encourage the encoder layers to find a mapping between the original input and a pathway-based latent representation of the sample in the middle layer. To ensure that the middle layer representation is still meaningful, we rely on the decoder portion of the network to reconstruct the original transcriptome. Thus we can compare the reconstruction with the input and evaluate whether our model is still capturing meaningful information in the latent representation. We hope that the revised Figure 1 and revised legends and text will address these issues for future readers.

4. - What is the justification to set the node number and layer number determined?

   The primary design of the neural network with regard to node number and layer numbers was arrived at simply by balancing a need to keep the total size of the parameter space reasonably small, while allowing enough opportunity for the

network to compress and decompress the transcriptome. Of course, a more systematic approach is needed, so thank you for this question.

We have assessed our architecture choices as part of a hyperparameter sweep, which includes sampling from parameters such as batch size, learning, rate as well as the number of layers and nodes. We have added new sections about the hyperparameter sweep to the methods, results, and discussions, including Figure S5. The assessment shows that our original choices were reasonable. We did not cherry pick the perfect set of parameters based on the sweep as our main model because of the risk of overfitting that could occur without the validation through a third validation dataset.

5. - I am wondering if there are very similar works published before to compare the performance directly, rather than simple comparison with simple autoencoders

   It would be an interesting comparison and some of the papers we cited in the introduction are comparable with regard to the goal of increasing the interpretability of the transcriptome. However, due to current trends, these methods are exclusively trained in the context of single-cell RNA data. The key feature of our paper is to show the feasibility of biological priors. Having established that in this paper, we look forward to extending the method to single cell data sets, and ideally integrating bulk and single cell RNA-seq together in a single latent representation. Such a molecular methods spanning model would then be directly comparable to other tools.

Reviewer #3:

Summary

This study develops an innovative approach to identifying biological processes undergoing perturbation from transcriptome data using variational autoencoders. The study achieves this through the incorporation of biological priors to direct the VAE networks to learn representations of transcriptomes that are based on biological concepts and could in principle be easier to interpret biologically. The authors show that both a simple fully-connected VAE and the novel prior-informed VAE can learn reduced representations of transcriptomes from high dimensions to 50 latent dimensions. These representations retain meaningful biological information that enable accurate reconstruction. While the fully connected VAE outperforms the prior-informed VAE in disease and organ classification tasks, it lacks directly interepretable latent dimensions. The prior-informed (beta-weighted) VAE not only solves the benchmark tasks but also provides semantically accurate latent features that map to biological pathways.

Overall, the work presented is innovative conceptually and methodologically. The main drawbacks include lack of rigor in benchmarking performance of the autoencoders as only a single metric (precision) is used to compare the models without consideration of the tradeoff to recall. There is need to assess the biological properties of the reconstructed transcriptomes related to the input datasets, especially an understanding of any relationships between reconstruction error and transcript levels of specific genes, pathways or tissue source/ disease state of the input samples. The ability of the prior-informed VAE to identify biological pathways that discriminate tissue source or disease state should also be compared to pathways that would be identified using conventional differential gene expression analysis and pathway enrichment. The validation of the prior-informed VAE's ability to enhance interpretability is weakened by the use of a set of 50-pathways as the source of priors for the latent space as the pathways are not comprehensive or representative of wide range of biological processes.

Strengths

-The approach to leverage VAE to identify differentially expressed biological pathways is innovative

-The approach addresses some key limitations of conventional approaches for differential gene expression analysis such as GSEA that require lists of well defined differentially expressed genes

-Unlike previous differential gene expression analysis approaches such as limma, DESeq2 and Seurat, the use of the prior-informed VAE does not assume linearity across samples

-The approach developed using autoencoders to enhance interpretability is distinct from other approaches that have been developed in the field using VAE. Unlike prior approaches that correlate phenotypes to latent features post-hoc or constrain the networks using known gene-pathway associations, the prior-informed VAE in this study enables the networks to leverage biological priors while still giving them freedom to learn relationships among genes

Major Limitations

1. -Authors need to perform a more detailed analysis of the samples where the correlation between the a given input transcriptome was not the most correlated to its reconstructed output to understand underlying mechanisms. This is to ensure future user can understand contexts in which the reconstruction may become unreliable

This comment ties together with the next several suggestions that the reviewer provided. Taken together, based on the reviewer's suggestions, we have expanded the analysis of the reconstruction performance in several ways. We have added Supplemental Figure S3, which provides an assessment of the gene-specific reconstruction error as a function of expression level. This shows that in fact there are some specific genes for which the error is exceptionally high. The highest error is associated with ribosomal RNA, which is not surprising since the levels of ribosomal RNA detected may have more to do with sample quality or sample preparation than the biology of the sample itself. Finally, another addition to the manuscript in the service providing transparency into the features of the reconstructed transcriptomes is that we provide t-SNE plots of original and reconstructed samples (Figure S4). Overall the clusters of the reconstructed samples are of a similar nature to the originals, and some level of difference is expected due to the stochastic nature of TSNE at the very least.

2. -There is need to assess any biases in reconstruction error at the gene and pathway level. For example, are there some genes or pathways whose transcript levels are more prone to higher error in reconstruction?

   Please see our response above. Gene-level reconstruction error can be found in plot S3. Regarding reconstruction at the pathway level, please refer to supplemental plots S6 and and b which indicate to what extent the pathway based priors are consistent with the model results of pathway representation.

3. -There is need to assess reconstruction performance based on highly vs lowly expressed genes as biases in these gene groups could impact biological outcomes typically based on gene expression

   This is an excellent idea and is now part of Figure S3.

4. -The tissue source/ treatment of the inputs need to be fully described in order to have a better understanding of the biological relevance of the results. Labeling the sample names with intuitive names will help in determining whether the observed correlations across tissues also capture tissue relationships

   Thank you for this suggestion. We have added an additional heatmap style plot reporting the input/output correlations and annotated with the tissue types (Figure S2).

5. -A comparison of the classification performance using differential gene expression or expression profiles of each sample directly would also be informative and useful to the field to show any advantages and disadvantages

In the updated manuscript, we have performed differential gene expression on the adeno vs SCLC dataset (Table 3), which provides a point of comparison between the autoencoder approach vs differential expressed gene approaches. We did not go so far as to build classification models based on DE genes. It is difficult to find an appropriate point of comparison here for the idea that you are after. Using a feature selection method like LASSO would be necessary and likely result in a selection of marker genes that are well suited to the classification task at hand. But this would be an apples and oranges comparison because the autoencoders are trained as generalized models and do not include anything comparable to a task-specific feature selection. More analogous to our approach could be to perform a PCA of the transcriptomes, and then build classifiers based on a certain number of top PCs. But please keep in mind that these classification tasks are just proxies for something that we do not have: ground truth about what basic biological processes are underway in the respective samples. We believe that what we do achieve with these benchmarks is a demonstration that enough biological information is still present in our compressed latent space, and the benchmarks also serve a point of comparison among the various architectures and design choices surrounding the Autoencoder method.

6. -Assessment of the performance needs to include recall and area under precision recall and the associated AUPRC curves. Without knowledge of the recall in relation to the precision, the utility of the high precision scores is low (including in the case where precision was 100%)

   We agree that our original metrics did not cover the full extent of what is necessary to report the performance of our models. Sorry for this. Our classification based benchmarks have all been updated to include precision, recall, accuracy, f1, and area under ROC.

7. -The results of differential analysis features across conditions needs to be compared to conventional approaches e.g. GSEA. For example, when comparing adenocarcinoma vs. health tissue, what are the differentially expressed pathways obtained using typical methods (e.g. GSEA)?

   Indeed this is quite an interesting comparison. We have performed differential expression analysis using limma and enrichment using EnrichR on KEGG pathways for our primary biological test case (adeno vs SCLC). We report the results in Table 3. It seems that the traditional method is more likely to pick up RNA degradation artifacts.

8. -The Pathways captured by the 50-dimensions are not comprehensive so it seems to be a big biological flaw to try to reduce the transcriptome dimensionality to these 50 pathways and use those results for interpretability. It would be better to use more comprehensive ontologies e.g. GO or pathways (KEGG) or to modify the prior informed VAE to include a dimension for "Unknown pathways"

Thank you for these suggestions. We have added this analysis and trained a model with selected KEGG pathways with and without unknown pathways using a similar procedure and process. The results can be seen in a new Figure 4. In fact the combination of KEGG + wild card was the best performing model in the tissue classification benchmark. Results and Discussion sections have been updated accordingly.

Minor Limitations

9. -It would be useful to show how well the network-based clustering that was performed to identify representative genes is representative of the full transcriptome

   This is true. The performance of our network based can be viewed in the new Figure S1, which addresses reconstruction of community level representation of the transcriptome. This task should be easier than reconstructing the full transcriptome, but even here we already see a somewhat poor performance when it comes to the reconstruction correlations for the beta models. As a result, our paper focused on the gene-level input for our models. It is possible that by adopting more sophisticated community level detection, those models could be competitive with gene-level inputs, and then this question of full reconstruction would be particularly important. This potential is now touched on in the discussion.

10. -It would be insightful if the heatmaps of the inputs and outputs were visualized separately in addition to as shown in Fig. 2. Separate visualization figure for inputs vs. outputs would help to demonstrate whether tissue correlations based on reconstructed data recapitulate the tissue correlations based on inputs. This could also be visualized using tSNE

    Thank you for the suggestion. We have now provided a few additional figures that should demonstrate the reconstruction with regard to tissues and with regard to the relationships among inputs separately from outputs. Please see Figure S2 for heatmaps labeled with tissues and Figure S4 for t-SNE Plots which depict the clustering of input samples as well as the clustering among reconstructed samples.

-Supplementary Fig. 1 is missing heatmaps for simple VAE and beta-simple VAE transcript associations

Thank you, we have rectified the issue (Figure S1).
